# Individualizing Surveillance after Endovascular Aortic Repair Using a Modular Imaging Algorithm

**DOI:** 10.3390/diagnostics14090930

**Published:** 2024-04-29

**Authors:** Amun Georg Hofmann, Irene Mlekusch, Georg Wickenhauser, Corinna Walter, Fadi Taher, Afshin Assadian

**Affiliations:** Department of Vascular and Endovascular Surgery, Klinik Ottakring, Montleartstraße 37, 1160 Vienna, Austria

**Keywords:** ultrasound, imaging, EVAR, endovascular aortic repair, FEVAR

## Abstract

Objectives: Surveillance after endovascular aortic repair (EVAR) and fenestrated EVAR (FEVAR) is mainly directed by one-size-fits-all approaches instead of personalized decision making, even though treatment strategies and often endografts themselves are tailor-made to adjust for individual patients. We propose a modular imaging algorithm that escalates surveillance imaging based on invasiveness and need. Materials and Methods: In this retrospective observational study of single-center data, results of a modular imaging algorithm were analyzed. The algorithm is characterized by initiating the examination with standard B-mode then transitioning to Duplex ultrasound, B-Flow, and CEUS. Additional CT(A) studies are conducted where required. The study population included both patients receiving EVAR or FEVAR. A comparative analysis was conducted regarding endoleak detection. Results: The study population included 28 patients receiving EVAR and 40 patients receiving FEVAR. They accounted for 101 follow-up visits, which led to 431 distinct imaging studies. CEUS has the highest endoleak detection rate, followed by CTA and B-Flow. Duplex ultrasound and B-Flow resulted in 0 and 1 false positive cases, respectively, considering CEUS the reference standard. In a select group of six patients, CEUS was omitted after endoleaks were displayed by Duplex ultrasound or B-Flow, leading to a successful type II coiling and no aneurysm-related adverse events. Conclusions: The proposed modular algorithm showed great potential to incorporate principles of personalized medicine in surveillance after endovascular aortic treatment. Since Duplex ultrasound and B-Flow rarely cause false positive endoleaks, more resource-intensive and invasive imaging studies such as CEUS and CTA can be omitted after positive identification.

## 1. Introduction

Endovascular aortic aneurysm repair (EVAR) has emerged as a valuable treatment option for infrarenal abdominal aortic aneurysms (AAA) since it was developed in the mid-1980s [1]. EVAR has several advantages to open surgical repair (OSR), including improved peri-operative survival [2], while absent long-term survival benefits, higher reintervention, as well as rupture rates [3] constitute comparative disadvantages. Treatment strategies are therefore developed in a personalized case-by-case manner [4]. In juxta- and pararenal aneurysms, treatment via fenestrated endovascular aortic repair (FEVAR) has undergone rapid popularization in recent years, as evidence by its number of reported cases in the published literature, which has now matched that of OSR [5]. FEVAR usually requires a custom-made stentgraft, as well as bridging stentgrafts to maintain blood flow to visceral target vessels [6]. Analogous to EVAR, it offers a treatment option for patients otherwise not fit for OSR [7], while also being associated with higher reintervention rates [8]. However, a meta-analysis questioned the survival benefit but discovered a favorable postinterventional association with lower morbidity rates [8].

The higher reintervention rates have several underlying etiologies, including in-stent thrombosis, mechanical complications associated with the prosthesis, and endoleaks [9]. Endoleaks are both the most common complication [10] as well as the most common indication for secondary intervention after EVAR [11]. Persisting feeding of the aneurysm sac can cause further AAA expansion and, in the worst of cases, induce subsequent rupture [12]. Accordingly, endoleaks constitute the most frequent cause of aneurysm rupture following EVAR [13]. Endoleak classification is based on their location and dysfunctional stent component ranging from type I to IV endoleaks, with endotension occasionally being referred to as a type V endoleak [4,14]. After FEVAR, endoleaks have a similar classification scheme that includes additional sub-classifications to account for leakage from renal or visceral target vessels [15].

The potential for prosthesis-related complications in general and endoleaks specifically requires continuous surveillance. A plethora of imaging modalities have been investigated historically. Computed-tomography angiography (CTA) is frequently discussed as the reference/gold standard after EVAR [14]. However, magnetic resonance imaging angiography (MRA) has been shown to be more sensitive for endoleak detection [16], while duplex ultrasound (DUS) has a high specificity but a low sensitivity when compared to CTA [17]. Contrast-enhanced ultrasound has both a high specificity and a high sensitivity regarding endoleak detection [18]. After FEVAR, the advantages of CTA for endoleak detection also established it as the preferred surveillance imaging modality [19]. Similarly, DUS and CEUS are frequently explored alternatives with adequate accuracy regarding endoleak detection [20]. CEUS has also been discussed to be superior to CTA due to its dynamic imaging attributes [21]. Additionally, CEUS has been repeatedly discussed to be a legitimate replacement for CTA during surveillance after endovascular aortic repair [22,23,24].

While the therapeutic strategy and, especially in the case of FEVAR, also the graft are individualized towards each patient, guideline-recommended surveillance strategies historically suggested a one-size-fits-all approach [4]. This changed partially in the most recent clinical practice guidelines of the European Society for Vascular Surgery, where, at least after standard EVAR surveillance imaging, intervals between imaging studies can be prolonged depending on the respective findings [25]. These recommendations relate to personalized surveillance regimens that have been previously explored but with their focus on the intervals between follow-up visits [26]; imaging modalities themselves are generally either ultrasound- or computed-tomography-angiography-based and, in practice, up to the surgeon’s or center’s discretion. In the present project, we investigated a modular imaging algorithm intended to further individualize surveillance strategies.

## 2. Materials and Methods

### 2.1. Ethics Statement

This study was approved by the Ethics committee of the City Government of Vienna (ID: EK-20-007-VK) and was conducted in compliance with the Declaration of Helsinki.

### 2.2. Patient Recruitment and Study Design

Following review board approval including a waiver of informed consent, a retrospective analysis of patients from a prospectively held database on endovascular aortic repair cases was performed. Only follow-up investigations including CEUS studies were included in the study. Patients without sufficient follow-up imaging data or those followed up by only CTA were excluded from the analysis. The total number of EVAR and FEVAR patients treated during the study period is given as a denominator, with hospital administration documentation accessed in addition to the prospectively held database for verification purposes. Most patients in the present study had ultrasound investigations due to impaired kidney function, allergy to iodinated contrast enhancers, to validate inconclusive CTA studies, or to monitor previously discovered endoleaks on short-frequency intervals and are, therefore, a select group of patients out of the entire patient pool. The regular EVAR and FEVAR follow-up protocol at our center is based on a postoperative CTA, a six-month CTA, and yearly CTA scans thereafter, as suggested by the current guidelines.

### 2.3. Computed Tomography

Computed tomography with or without angiography were performed by a radiologist either in-house or in external institutes. Computed tomography results without angiography were only used to study aneurysm diameters. CTAs were performed as a triple-phase study including unenhanced, arterial contrast-enhanced, and delayed phases. CTA study reviews for this investigation were routinely performed by radiologists and re-examined by a vascular surgeon in the outpatient clinic. Endoleaks were classified according to the current guidelines of the European Society of Vascular Surgery [4].

### 2.4. Ultrasound Examinations

Ultrasound examinations were performed using the Logiq S8 device (GE Healthcare, Chicago, IL, USA). Aneurysm diameter was exclusively measured in B-mode. CEUS was performed after intravenous Sonovue (Bracco, Milano, Italy) injection as a bolus followed by a flush of saline solution. Images and clips were locally stored on the ultrasound devices.

### 2.5. Modular Imaging Algorithm

**Figure 1:**
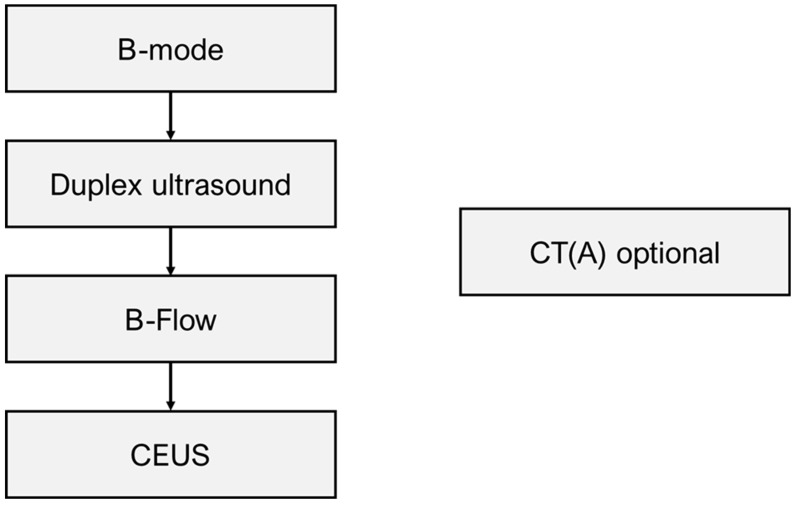
Flowchart depicting the modular imaging workflow used to conduct surveillance studies. (CEUS = contrast-enhanced ultrasound, CT(A) = computed tomography with or without angiography).

The applied imaging algorithm is depicted in Figure 1. Ultrasound studies were initiated in B-mode to investigate the aneurysm diameter as well as morphological features of the aneurysm sac. In general, B-mode was followed by DUS, B-Flow, and CEUS. Examiners are therefore not blinded to previous modules and can decide based on the obtained information whether to progress. CT(A) was additionally conducted at the examiner’s discretion where deemed necessary and feasible. Potential reasons included diverging results in different ultrasound modalities; limited investigation conditions, for example, due to bowel gas; or planning of indicated reinterventions. The proposition of this workflow is based on the increasing sensitivity regarding endoleak detection in the published literature, with CEUS serving as the reference standard for the present study due to its reported high accuracy.

### 2.6. Statistical Analysis

Statistical analysis was performed with R version 4.1.3 (One Push-Up; The R Foundation for Statistical Computing, Vienna, Austria) in RStudio version 2022.02.2+485 (RStudio, PBC, Boston, MA, USA). Descriptive statistics were conducted using standard methodology. Averages are depicted as median (1st–3rd quartile) or mean (+/− standard deviation); proportions are given in percent. Agreement for endoleak classifications is expressed in percent, while reliability (strength of agreement) is assessed using Cohen’s kappa. For this study, we included 2 patients receiving BEVAR and 1 patient receiving an F/BEVAR hybrid graft in the FEVAR subgroup.

## 3. Results

### 3.1. Participant Characteristics

In total, 68 patients were included, out of which 40 received FEVAR (including 4 with FEVAR after previous EVAR) and 28 underwent standard EVAR. Additionally, 13 patients received a TEVAR to treat pathologies extending into the descending aorta. The sample consists of 62 men and 6 women. Dyslipidemia was the most prevalent comorbidity (77.9%), followed by hypertension (75.0%) and chronic kidney disease (72.1%). Primary procedures were conducted between August 2009 and May 2023. Patients underwent a total of 101 follow-up appointments, resulting in 410 imaging studies. The follow-up examinations were conducted between December 2017 and July 2023. Median time since the index procedure was 745 days (Q1–Q3: 161–1371 days). Participant characteristics are shown in Table 1. Due to missing data, the 101 conducted CEUS studies had 83 paired studies with prior DUS and B-Flow examination, 7 with a previous B-Flow investigation only, and 3 with a prior DUS. The remaining eight visits included five with a documented paired CTA study. The missing data were attributed to lack of electronic documentation.

### 3.2. Aneurysm Diameter

**Figure 2:**
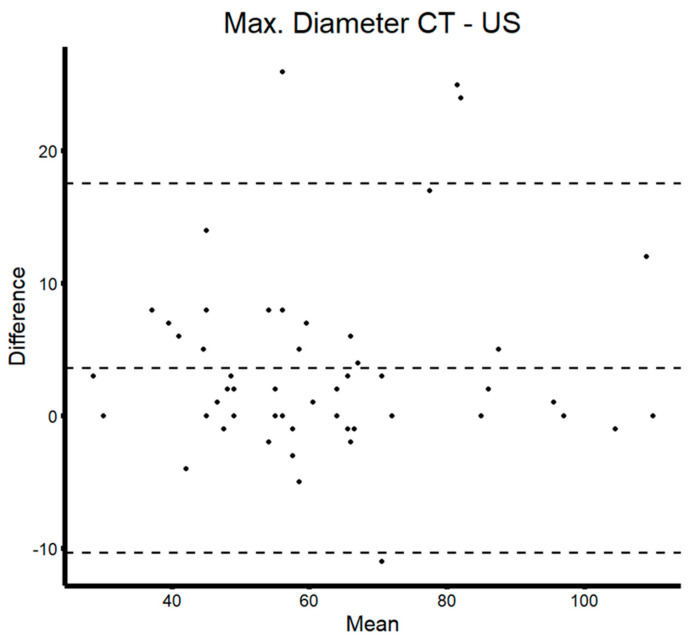
Bland–Altman plot of maximum aneurysm diameters measured in CT(A) and ultrasound.

As a quality-control step, maximum aneurysm diameter, as measured by ultrasound and CT scan, was compared to ensure validity of the conducted ultrasound studies. In the present sample, 45 paired CT or CTA and ultrasound examinations were included. The median delay between CT and ultrasound was 42 days (Q1–Q3: 6–76 days). Mean difference between imaging modalities was less than 4 mm and, except for four studies, was found to be within the confidence interval of the Bland–Altman plot (see Figure 2).

### 3.3. Endoleak Detection Rates

**Figure 3:**
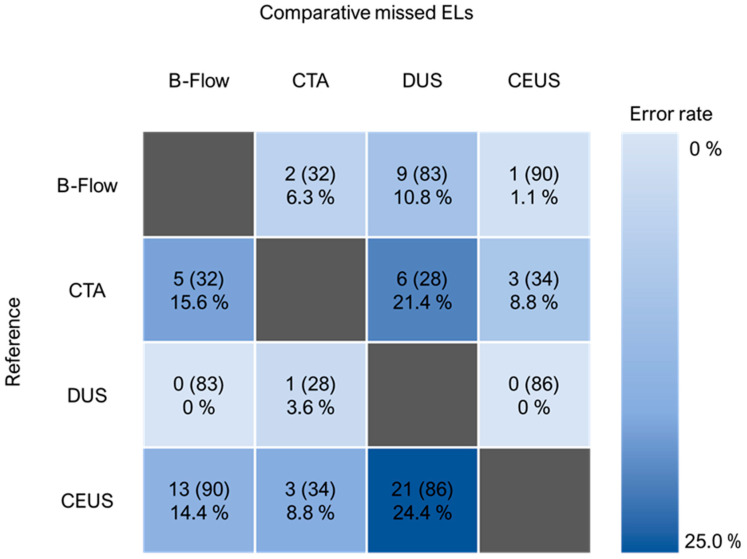
This figure displays how many endoleaks would have been missed in a specific imaging modality (reference) compared to paired investigations with other technologies. Number in brackets is the sum of endoleaks found in the reference imaging modality in paired studies. Percentage points display the relative error rate in paired investigations. (ELs = endoleaks).

CTA identified 26 endoleaks in 34 conducted studies, while DUS revealed 24 in 86, B-Flow displayed 38 in 90, and CEUS 56 in 101 documented ultrasound studies. To further ensure the validity of our modular imaging workflow, we investigated whether endoleak detection rates would increase with each subsequent step. Comparing the error rates of the different imaging modalities, in our sample, CEUS showed the lowest error rates on average, followed closely by CTA. However, the availability of paired CTA studies is also limited compared to paired CEUS investigations and, therefore, associated with a higher statistical uncertainty. B-Flow missed less than 15% of the endoleaks found in CTA and CEUS, while, in the case of DUS, over 24% of endoleaks visualized by CTA and CEUS could not be identified (all see Figure 3).

Regarding endoleak classifications we observed that ultrasound studies had a very high agreement with each other (74.4–88.0%). Since agreement does not correct for the incidence rate of an event, a reliability metric (Cohen’s kappa) was additionally calculated and assessed (see Table 2). The strength of agreement between B-Flow and both CEUS and DUS was good, while the recommended interpretation of Cohen’s kappa for CEUS and DUS was moderate. However, this includes missed endoleaks, which means that an endoleak not identified in one modality but displayed in another will also negatively affect agreement and reliability metrics between the two. After excluding missed endoleaks DUS and CEUS as well as DUS and B-Flow had 0 disagreeing classifications, while CEUS and B-Flow had one differently classified endoleak (type I in B-Flow; type II in CEUS). With a smaller sample size, agreement and reliability were found to be lower with CTA and all ultrasound-based modalities.

### 3.4. Evaluation of the Modular Algorithm

The main proposed advantage of a modular imaging algorithm is the case-specific decision to conduct resource-expensive investigations depending on prior studies while maintaining a safe and accurate surveillance program. If an endoleak can be detected in DUS or B-Flow, further investigation with CEUS should become redundant. However, omitting this subsequent diagnostic step is only feasible if the detected endoleaks are correct identifications. Therefore, we investigated the rate of false positives in DUS and B-Flow. DUS had no false positive endoleak detection, while B-Flow had one. The false positive prediction rates and associated 95% confidence intervals suggest that DUS and B-Flow have a low probability of producing high false positive endoleak rates compared to CEUS as a reference standard (see Table 3).

### 3.5. Forgoing CEUS in Selected Cases

In a small validation cohort of six cases, endoleaks were detected using B-Flow; in four of those, the endoleak was visible already using DUS. Three cases had a previous CTA that already displayed the endoleak. In these cases, the endoleaks were clearly displayed to three different observers and, therefore, the decision was made to forgo additional CEUS. These follow-ups happened between July 2021 and January 2023. One of the patients underwent successful interventional angiography after the B-Flow depicted type II endoleak. The remaining patients had no stentgraft-related adverse events in the aftermath of the respective follow-up appointment. In this limited sample of selected cases, omitting CEUS after positive endoleak detection in B-Flow had positive preliminary results.

## 4. Discussion

Ultrasound is a valuable and accurate technology for surveillance after EVAR and FEVAR and there are no indications that it has significant disadvantages to CTA. While certain questions require at least a computed tomography without angiography, such as evaluation of the proximal landing zone, especially in FEVAR or T-/FEVAR cases, the most important aspects can be investigated via sonography. This includes both aneurysm diameter assessment and (associated) endoleak detection. The present results illustrate that ultrasound-based imaging studies lead to reliable aneurysm sac diameters as compared to computed tomography imaging, arguably one of the most important features of post (F)EVAR surveillance.

In our sample, CEUS also showed the highest endoleak detection rate, indicating advantages of dynamic imaging modalities and further boosting the cumulative body of evidence that suggests CEUS as the reference standard for surveillance after EVAR and FEVAR. Regarding EL detection after EVAR, CEUS has repeatedly been found to be at least noninferior [27] or even superior [28] to CTA. Endoleak type classifications also showed very strong agreement between all ultrasound-based technologies, while CTA resulted in several disagreeing classifications. However, CTA does not inform the observer about the direction of flow, giving it an inherent disadvantage regarding classifications. Our results also show that the subsequent use of DUS, B-Flow, and CEUS in that order is reasonable considering the increased rate in EL displays. Since DUS and B-Flow are at low risk of producing false positive results, endoleak detection using these imaging modalities can be regarded as true endoleaks and might not need further investigation with CEUS or CTA. However, CTA may be necessary for further treatment planning.

The potential issues associated with contrast enhancers [29] or cumulative exposure to radiation and associated cancer risk [30] are considerable limitations of CTA. Contrast enhancement needed for CEUS has less limitations but can still induce anaphylactic shock and requires intravenous application [31].

We initiate imaging with standard B-mode for aneurysm diameter measurement and morphological assessment to then transition to DUS, B-Flow, and, finally, CEUS. This workflow reflects the increasing accuracy of the respective ultrasound modes regarding endoleak detection. We developed and applied this imaging algorithm as part of our routine endovascular surveillance protocol. In the present analysis, we included patients that underwent each module to gather preliminary evidence. However, it remains to be investigated whether exiting the algorithm after endoleak identification in DUS or B-Flow is associated with impaired outcomes compared to patients who continuously receive CEUS investigations as the reference standard. While we explored this in a limited set of patients without any adverse outcomes, the level of evidence is still too low to provide final conclusions.

B-Flow is a technology developed by GE and, therefore, only present in GE devices [32]. We have recently shown that it is feasible to detect endoleaks after EVAR and FEVAR using B-Flow and, compared to DUS and CEUS, its endoleak detection rate is just in between the two [33,34]. However, in the absence of B-Flow, our imaging algorithm can still be applied by removing the B-Flow module and transitioning from DUS to CEUS when necessary. Recently, superb microvascular imaging (SMI), a technology developed by Toshiba, has also been investigated for surveillance after EVAR with satisfying results [35]. It seems reasonable to assume that the B-Flow module can be exchanged for SMI when available. Where deemed necessary and feasible, additional CTA studies can be used to complement the algorithm. However, as evidenced by our data and previously published works, CTA is not necessarily more accurate than CEUS for endoleak identification. In the absence of aneurysm sac enlargement (or ideally if aneurysm sac shrinkage is observed), a type II endoleak detected by CEUS may thus be sufficient documentation for the respective surveillance timepoint without a need for subsequent CTA. Since CTA may be required for planning reinterventions, an obvious type I or III endoleak detected in DUS (or B-Flow) may still allow the subsequent B-Flow or CEUS diagnostic step to be omitted in favor of a CT scan. This requires further investigation in larger scale and potentially multi-center and prospective trials.

Proposed advantages of such a modular imaging algorithm are an improved cost-effectiveness by only conducting more resource-intensive studies (CEUS or CTA) when necessary and a reduced risk for adverse events induced by ionizing radiation (CTA) or anaphylactic shock after intravenous contrast enhancer application (CEUS and CTA) by forgoing these imaging studies when feasible. Health economic factors are of increasing interest regarding surveillance after (F)EVAR and, recently, both a dedicated CEUS-only approach [36] as well as a hybrid CTA/DUS strategy [37] have been proposed as the most cost-effective methods. However, negative findings in the early modules require subsequent imaging studies, thereby prolonging follow-up visits compared to protocols that base surveillance on CTA or CEUS only. Combined with the fact that adequate reimbursement by health insurances for follow-up ultrasound studies after (F)EVAR can be difficult (especially when conducting multiple ultrasound studies) depending on the geographic location, a modular imaging algorithm might also introduce economic pressure.

Modular imaging algorithms can already be an effective approach to transition from a competition aimed at identifying the best-suitable imaging technology by acknowledging distinct benefits and limitations and counterbalancing them based on availability and need. A further improvement regarding the combined use of different imaging modalities might be achieved by ultrasound image fusion with CT(A). This has been previously investigated as an effective strategy [38,39], has the potential to significantly improve surveillance after endovascular aortic repair, and can also be implemented in a modular imaging algorithm to be used in selected cases when required.

### Limitations

Several limitations apply to the current study. The sample size is too small to gather conclusive evidence. The single-center setting and composition of the study cohort introduce a further risk for selection bias. However, it is appropriate to base further trials on the collected data and use it as the foundation for future investigations to individualize and optimize follow-up protocols after endovascular aortic interventions. The generalizability of this study might also be limited due to resource factors such as the availability of trained vascular ultrasound technicians or patient factors such as increased rates of obesity in certain geographic locations that limit the effectiveness of ultrasound investigations. Even in environments where standardized ultrasound-based surveillance studies are feasible after endovascular aortic repair, TEVAR or T/FEVAR might impose further restrictions. Adequate investigation of the proximal sealing zone using ultrasound only is unachievable and further studies are warranted to optimize imaging algorithms for pathologies extending the visceral segment. Additionally, while several research efforts such as the present one have aimed at limiting the frequency of CT(A) studies during surveillance, it should be noted that, even though inter- and intra-observer differences in ultrasound-based studies are acceptable, they do not offer the reproducibility of CT(A) scans.

## 5. Conclusions

While the present analyses might need further spin-off studies to generate conclusive evidence, they show promise for a transition to precision medicine in surveillance after endovascular aortic treatments. Future trials could combine a prospective design with an adequate sample size deducted from our pilot data and further standardize CTA indications as well as validate findings in a core lab.

## Figures and Tables

**Table 1:** Demographic and clinical characteristics of the study population. Age given as median (Q1–Q3).

	EVAR	FEVAR	Total
Number of patients	28	40	68
Sex (female:male)	2:26	4:36	6:62
Age (at surveillance)	80.1 (69.7–82.2)	77.3 (71.2–81.2)	77.5 (70.4–82.3)
Follow-up appointments	37	64	101
*Imaging studies*	167	264	431
CT(A)	22	33	45
B-mode	37	62	99
DUS	35	51	86
B-Flow	36	54	90
CEUS	37	64	101
FEVAR after EVAR	-	4	4
Target vessels	-	136	136
Additional TEVAR	-	13	13
Chronic Kidney Disease	16	33	49
Arterial Hypertension	20	31	51
Coronary Heart Disease	12	28	40
Dyslipidaemia	23	30	53
Diabetes	5	11	16
Chronic obstructive pulmonary disease	11	17	28

**Table 2:** Agreement (in %) and reliability (Cohen’s kappa) metrics evaluating endoleak classifications of paired imaging studies.

	B-Flow	DUS	CEUS	CTA
B-Flow	-	88.0%	82.2%	65.6%
DUS	0.748	-	74.4%	60.7%
CEUS	0.690	0.519	-	61.8%
CTA	0.432	0.325	0.355	-

**Table 3:** Metrics evaluating false positive detections of DUS and B-Flow compared to CEUS.

	False Positives	False Positive Rate	95% CI
DUS	0	0%	0–4.4%
B-Flow	1	1.1%	0–6.0%

## Data Availability

Data can be made available upon reasonable request to the corresponding author.
